# Patient Perceptions on the Use of Artificial Intelligence in Creating Clinical Research Documents: Survey Study

**DOI:** 10.2196/76547

**Published:** 2026-06-22

**Authors:** Kimbra Edwards, Samuel Entwisle, Zack Fey, Hana Do, Art Gertel, Annick de Bruin, Kenneth Getz

**Affiliations:** 1 Center for Information and Study on Clinical Research Participation, One Liberty Square, Suite 1100, Boston, MA, 02109, United States, 1 617-725-2750 ext 118; 2 Tufts Center for the Study of Drug Development, Tufts University School of Medicine, Boston, MA, United States; 3 MedSciCom, Lebanon, NJ, United States

**Keywords:** artificial intelligence, patient engagement, clinical research, health communication, plain language summary

## Abstract

**Background:**

The use of generative artificial intelligence (AI) by pharmaceutical companies and other organizations for preparing patient-facing documents reporting results of clinical research is becoming more common. This raises concerns about whether the accuracy and quality of these documents could be affected, as well as the potential impact on patient perceptions and trust. Accurate and trustworthy information is critical to health care decision-making. Little is known about patient perceptions of AI-generated content.

**Objective:**

This study aimed to better understand patient experience and familiarity with AI, their resulting confidence in the abilities of AI, and their trust in the use of AI by research organizations to generate clinical research documents.

**Methods:**

An online survey was conducted using an online health care panel of patients in Europe and the United States to assess familiarity with AI, trust in organizations reporting on research, and trust in the use of AI to prepare clinical research documents. The survey also asked directly about the importance of human involvement and of transparency in disclosing AI use.

**Results:**

A total of 1010 respondents completed the online survey. About half of respondents were from the United States and half from Europe. Survey results showed that 63.6% (642/1010) of respondents had used AI before with 74.9% (756/1010) reporting being “Somewhat” or “Very” familiar with AI. AI use was influenced by country, gender, education level, race/ethnicity, and clinical trial experience. Higher familiarity with AI was observed among younger participants. Respondents were generally confident in the capabilities of AI, as more than half believed AI use would reduce grammar and data errors. Trust in clinical trial documents generally increased with greater human oversight, as trust was lowest for documents created by AI with no human involvement (12.3% “A lot” of trust, 124/1010) and highest for documents created by humans without AI (39.1% “A lot” of trust, 395/1010). 95.0% (959/1010) of respondents considered human involvement in clinical trial document review as “Very important” or “Somewhat important.” The majority (62.4%, 630/1010) of respondents felt it was “Very important” for pharmaceutical companies and academic institutions to be transparent about their use of AI in public-facing documents. Transparency was considered more important among respondents in the United States and the United Kingdom compared to those in the European Union.

**Conclusions:**

The survey results reveal high familiarity with, and confidence in the capabilities of, AI. Despite this confidence, respondents emphasized the need for human involvement in the creation of clinical trial documents and the importance of disclosing AI usage, underscoring the critical role of human oversight in maintaining patient trust. Transparent integration of AI with deliberate human involvement remains essential to ensure trust in patient-facing documents.

## Introduction

### Generative Artificial Intelligence in Health Communication

The use of generative artificial intelligence (AI; AI that can create new content including images and text) in clinical research and health communication has become increasingly prevalent [1-3]. Health technologies that use AI, such as chatbots, are being developed to provide personalized health care support in a variety of areas including obesity, diabetes, smoking cessation, and mental health [4].

One increasingly common use of AI is in the creation of patient-facing documents [5,6]. Plain language summaries of clinical trial results, which are mandatory by the European Union (EU) Clinical Trial Regulation 536/2014 for sponsor companies conducting trials among patients in EU-member countries, may be a particularly attractive target for AI support due to their structured nature and high volume. However, while AI has the potential to streamline the creation of patient-facing documents, its use must be approached with caution, particularly given the need for clear, unbiased, and culturally appropriate communication in health care. Patients and the public, especially those of certain races or ethnicities, often view health care systems, clinical research, and pharmaceutical companies with skepticism [7-10], and any misstep in how information is conveyed could exacerbate this mistrust.

### Trust and Acceptance

Several studies have aimed to understand the factors that lead to increased acceptance of new AI-based health technologies [11,12]. However, current research about patient acceptance of AI use has mostly focused on its use in creating personalized care, rather than clinical research documents that have been created using AI. Likewise, theoretical frameworks for understanding adoption of new technologies such as the Technology Acceptance Model (TAM) [13] or the Unified Theory of Acceptance and Use of Technology (UTAUT) [14] may have limited utility in cases where the patient is not directly interacting with the AI technology, but rather is made aware of its prior use in developing the documents. It will be important to better understand patient and public perceptions about the use of AI in key documents related to clinical research and health care so that AI can be employed thoughtfully and effectively in these settings.

Patient and public involvement is often used to guide clinical development, and patient and public input is increasingly being gathered regarding the use of AI in clinical settings [15]. For example, a series of workshops allowed patients and members of the public to provide feedback about the use of generative AI in new technology for helping to manage asthma [16]. Similar to the use of AI in clinical care, patients and the public should also guide the implementation of AI in document creation, especially when preparing materials intended for the public. Guidelines exist for the use of AI in medical publications and publication ethics [17,18]. However, research on patient and public perceptions of AI-generated documents related to health care and clinical research remains limited [19].

### The Present Study

We surveyed patients to better understand their current perceptions of AI when used to help create clinical research documents. We hypothesized that:

- Familiarity with and confidence in AI are linked
- Familiarity with and confidence in AI vary based on demographic factors
- Human involvement leads to increased levels of trust compared to generation of clinical trial documents by AI alone

By directly surveying patients, we hope to gain a better understanding of the factors that impact patient trust in AI when used in the development of clinical trial documents. This will help to guide the development of future AI applications in this area.

## Methods

### Design

This survey (Multimedia Appendix 1) was organized by the Center for Information and Study on Clinical Research Participation (CISCRP), a nonprofit organization that aims to educate patients and the public about clinical research. In support of its mission, CISCRP partners with clinical research sponsors to provide health communication services, including patient- and public-facing clinical research documents. The survey was conducted via the Alchemer online platform to gather data on demographics, perceptions of AI-generated content, familiarity with AI, and trust in the pharmaceutical industry in March 2024. The survey was in English and took an average of six minutes to complete. It explored a primary theme of trust, as well as perceived accuracy, the importance of disclosing AI use, participants’ concerns, perceived benefits associated with the use of AI in clinical research document creation, and the impact of human involvement.

### Recruitment

We targeted individuals (18 years or older) with medical conditions and varying degrees of clinical research experience to ensure a diverse sample representing both individuals familiar and unfamiliar with clinical trials.

An online health care panel was engaged for this survey which included individuals across the US and Europe. The online health care panel enabled us to target specific regions and achieve balanced representation in terms of demographic variables and conditions. The survey was administered in English only and therefore countries where English is more prevalent were included. The panel was comprised of prerecruited groups of individuals who agreed to participate in surveys or interviews over a period of time. The response rate was 22.7% (1,010 surveys completed out of 4457 surveys sent). We prevented duplication using IP addresses by enabling features on the survey platform that blocked multiple submissions from the same IP address.

### Analysis

Data cleaning and analysis were conducted using R statistical software package (version 4.3.3; R Foundation for Statistical Computing). As respondents were required to complete all fields and questions to submit the survey, there were no missing data. Descriptive statistics, and appropriate statistical testing (Spearman’s rank order correlation or *t*-tests for continuous variables and *χ*^2^ for categorical variables) for relationships between variables of interest were performed. Bonferroni corrections were used to adjust for multiple testing. Subgroup analyses were performed across various demographics and characteristics, including region, age, gender, race, education, clinical trial experience, and trust in pharmaceutical companies and academic institutions. Some categories were merged for certain subgroup analyses; for example, survey respondents who selected “Unknown” and “No” clinical trial experience were combined. Where comparisons across subgroups were feasible and appropriate, *P* values were reported; *P*<.05 was considered significant. Responses to open-ended questions were reviewed and summarized using a descriptive approach to identify common ideas; however, no formal qualitative coding or thematic analysis was conducted.

### Ethical Considerations

All individuals in the panel opted in to be contacted for survey opportunities and were compensated $10 (or local currency equivalent) for completing the survey. The survey was deemed Institutional Review Board exempt by WCG Institutional Review Board and General Data Protection Regulation-compliant; all respondents consented to having their data collected and stored. The privacy policy of CISCRP was also available for reference. Completion of the survey posed minimal risk, the dataset was anonymized, and results were reported in aggregate with limited open-ended questions.

## Results

### Demographic Characteristics

Table 1 summarizes respondent characteristics. In all, we received 1010 completed responses from patients with approximately half based in Europe and half in the United States (Table 1).

**Table 1.** Demographics of survey respondents.

Demographics	Value (N=1010), n (%)
Age (years)	
18‐34	307 (30.4)
35‐44	208 (20.6)
45‐54	188 (18.6)
55‐64	156 (15.4)
65+	151 (15.0)
Gender	
Male	506 (50.1)
Female	482 (47.7)
Other^a^	19 (1.9)
Prefer not to answer	3 (0.3)
Race	
White	835 (82.7)
Black	75 (7.4)
Asian	31 (3.1)
American Indian or Alaska Native	13 (1.3)
Native Hawaiian or Pacific Islander	4 (0.4)
Mixed^b^	25 (2.5)
Other	27 (2.7)
Ethnicity	
Hispanic or Latino	204 (20.2)
Not Hispanic or Latino	693 (68.6)
Other	76 (7.5)
Prefer not to answer	37 (3.7)
Country	
United States	500 (49.5)
United Kingdom	92 (9.1)
Germany	90 (8.9)
Italy	90 (8.9)
Spain	89 (8.8)
France	89 (8.8)
Netherlands	60 (5.9)
Clinical trial experience	
Yes	243 (24.1)
No	734 (72.7)
Unknown	33 (3.3)
Education	
No Bachelor’s degree	618 (61.2)
Bachelor’s degree or higher	386 (38.2)
Prefer not to answer	6 (0.6)
Medical condition**^c^**	
Cardiometabolic and Endocrine	451 (44.7)
Musculoskeletal and Pain	488 (48.3)
Neurological and Behavioral	545 (54.0)
Immune, Infection and Hematologic	553 (54.8)
Gastrointestinal, Renal and Pulmonary	238 (23.6)
Specialized Health	712 (70.5)
Other	48 (4.8)

a Gender identities included in other: transgender male, transgender female, variant/non-conforming, and other (write in option).

b Mixed category contains respondents who selected more than one race.

c Medical conditions included in each category: Cardiometabolic & Endocrine: diabetes, endocrine/hormone, metabolism, heart/cardiovascular; Musculoskeletal & Pain: arthritis, musculoskeletal, pain; Neurological & Behavioral: neurology, headache/migraine, mental health; Immunologic, Infection, & Hematological: allergies, immune condition, infection, blood; Gastrointestinal, Renal & Pulmonary: gastrointestinal, kidney/bladder, lung; Specialized Health: sleep, eye, skin, cancer, female sexual health, male sexual health; Other: write in option. Survey respondents could have selected more than one condition.

### Familiarity with AI and Clinical Trial Documents

Respondents were asked whether they had used AI before (“Yes” or “No”), and about their familiarity with AI using a 4-point Likert scale. We found that 63.6% (642/1010) of respondents said that they had used AI before (Table S1 in Multimedia Appendix 2), and 74.9% (756/1010) of respondents said that they were “Somewhat” or “Very” familiar with AI. There was a significant relationship between country (*P*<.001)*,* gender (*P*<.001), education level (*P*=.002), race/ethnicity (*P*<.001), and clinical trial experience (*P*<.001) in terms of having used AI before (Table S1 in Multimedia Appendix 2). Some reported uses for AI included image generation, work-related tasks, homework help, and to answer general information questions. Younger respondents were significantly more likely to be familiar with AI (*P*<.001). The median age of respondents indicating they were “Very familiar” was 36 (IQR 28-45), while the median age of respondents indicating they were “Not at all familiar” was 55 (IQR 39-63).

Respondents also reported which, if any, clinical trial documents they had seen before, where options included informational brochures, informed consent documents, clinical trial protocol synopses, trial results summaries, and publication summaries. Among respondents, only 23.7% (239/1010) said that they had not seen any of the listed documents before, indicating at least a passing familiarity with clinical trial documents among most, despite the fact that 72.7% (734/1010) of respondents indicated they did not have clinical trial experience (Table 1). The majority of respondents (77.7%, 785/1010) were either “Somewhat” or “Very” interested in reading clinical trial documents that had been created at least in part by AI.

### Perceptions of AI and the Pharmaceutical Industry

More than half of respondents believed AI use would reduce grammar and spelling errors (58.0%, 586/1010) and data errors (57.6%, 582/1010). Additionally, respondents were asked to rate on a 4-point Likert scale whether they agreed with the statement that “AI always produces accurate and appropriate text and images.” While most respondents perceived AI as consistently producing accurate and appropriate content, this perception was more common among younger respondents, and opinions also varied across regions. About two-thirds of respondents (66.9%, 676/1010) said that they “Agree” or “Strongly agree” that AI always produces accurate and appropriate text and images. Similar to familiarity with AI, age had a significant effect on how participants responded (*P*<.001). The median age of respondents indicating they “Strongly agree” was 38, while the median age of respondents indicating they “Strongly disagree” was 59. In addition, respondents from EU countries were significantly more likely to say that they “Agree” with this statement (62.7%, 262/418) than the overall group (*P*<.001), and respondents from the US were significantly less likely to say that they “Agree” (46.6%, 233/500, *P*<.001). Non-White or Hispanic respondents were significantly more likely to say that they “Strongly agree” with this statement (18.2%, 76/417*; P*<.001) compared to White and non-Hispanic respondents (9.8%, 58/593, *P*<.001). In general, the more familiar respondents reported that they were with AI, the more likely they were to say that they “Agree” or “Strongly agree” that AI always produces accurate and appropriate text and images (*P*<.001).

Respondents were also provided with open text boxes to write any perceived value or fears of AI being used for the creation of clinical trial documents for patients and the public. Responses for perceived value varied dramatically, with some acknowledging AI’s potential to save time, organize data, and reduce errors, while others saw little to no value in AI being used in this capacity. For example, one respondent noted, “It will probably mean less errors long-term and save time and money,” and another wrote, “It has a lot of value. AI will save time if it is done in the right way.” However, others wrote, “Little value” or “none.” Responses for perceived fears were also varied, with some expressing no fears and others fearful of AI’s potential to introduce errors, spread misinformation, compromise data privacy, and replace human jobs. For example, one respondent wrote, “I really don’t have any fear,” and another mentioned, “No fears come to mind.” However, others wrote about their fears of “privacy,” “data breaches,” “lack of accuracy,” “room for error or misinformation,” “the fear of replacing human jobs,” and “no emotional factor.”

The majority of survey respondents indicated that they had at least some trust of pharmaceutical companies and academic institutions that conduct clinical research, as 50.8% (513/1010) of respondents said they had “Some” trust and 28.1% (284/1010) said that they had “A lot” of trust based on a 4-point Likert scale. Of all respondents, 77.0% (778/1010) believed that “A lot” or “Some” pharmaceutical companies and academic institutions are currently using AI to write patient- and public-facing clinical trial documents based on a 4-point Likert scale. Respondents who said that they have “A lot” of trust in pharmaceutical companies and academic institutions were more likely to respond that they had used AI before and that they “Strongly agree” that AI “always produces accurate and appropriate text and images” (*P*<.001).

### Trust in AI in the Context of Clinical Trial Documents

Respondents were asked about their level of trust (4-point Likert scale) in hypothetical documents that were drafted with varying levels of AI involvement. The survey results indicate that trust in clinical trial documents was tied to human involvement in their creation and oversight. Trust in clinical trial documents was lowest when written solely by AI without human involvement, with only 43.4% (438/1010) expressing “A lot” or “Some trust,” and 24.0% (242/1010) expressing no trust (Table 2). The highest trust levels were observed when documents were written exclusively by humans with 85.6% (865/1010) of respondents expressing “A lot” or “Some” trust and only 14.4% (145/1010) reporting “A little” or no trust; whereas 74.7% (754/1010) of respondents expressed “A lot” or “Some” trust and 25.3% (256/1010) reported “A little” or no trust when documents were written using AI with human review.

**Table 2.** Trust levels in clinical trial documents based on AI and human involvement in writing.

Amount of human involvement in clinical trial document writing	Trust level, n (%)			
	A lot	Some	A little	None
Written using AI^a^, without any human review	124 (12.3)	314 (31.1)	330 (32.7)	242 (24.0)
Written using AI, with human review	337 (33.4**)**	417 (41.3**)**	196 (19.4**)**	60 (5.9**)**
Written by humans, with no AI involvement	395 (39.1**)**	470 (46.5**)**	108 (10.7**)**	37 (3.7**)**

a AI: artificial intelligence.

Regardless of factors such as age, ethnicity, or familiarity with AI, respondents consistently demonstrated higher levels of trust with higher amounts of human involvement in writing.

Compared to those without prior clinical trial experience and a lower level of general trust in pharmaceutical companies and academia, respondents with prior clinical trial experience and higher general trust in pharmaceutical companies or academic institutions were more likely to trust AI-generated documents without human oversight; however, it should be noted that trust was significantly higher when documents were created solely by humans (*P*<.001). Specifically, 25.5% (62/243) of those with clinical trial experience and 26.1% (74/284) of those with high institutional trust reported “A lot” of trust in AI-authored documents without human review, compared to only 8.4% (62/734) of those with no clinical trial experience and 3.3% (7/213) of those with little or no institutional trust.

There were notable age-related differences in trust associated with the level of human involvement. Older respondents were significantly less likely to trust a document written using AI without any human review (*P*<.001). The median age of those who reported a trust level in such documents of “None” was 51 (IQR 35-63), whereas the median age of those who reported a trust level of “A lot” was 39 (IQR 29-44). Furthermore, the median age of respondents who reported it was “Very important” for humans to be involved in the review and development of these documents was 46 (IQR 33-60). In contrast, the median age of respondents who felt it was “Not at all important” was 34 (IQR 27-50).

### Importance of Human Involvement in Clinical Trial Documents

The vast majority of respondents (95.0%, 959/1010) considered human involvement at least “Somewhat important” (4-point Likert scale) in the review and development of patient-facing clinical trial documents. Specifically, 67.0% (677/1010) of respondents indicated that human involvement was “Very important,” while 27.9% (282/1010) rated it as “Somewhat important.” Only a small proportion of respondents felt it was less essential, with 3.3% (33/1010) considering it “Not very important” and 1.8% (18/1010) indicating it was “Not at all important.”

EU respondents were less likely than US and UK respondents to say that it was “Very important” (*P*<.001), and more likely than US and UK respondents to say that it was “Somewhat important” *(P*<.001) to have human involvement in document review and development.

### Importance of Disclosure of AI Use

Almost all respondents (92.9%, 938/1010) reported that it was at least “Somewhat important” (4-point Likert scale) for pharmaceutical companies and academic institutions to make it clear if, and how, they have used AI in public- and patient-facing documents, with 62.4% (630/1010) reporting that it was “Very important.” Only 5.6% (57/1010) felt it was “Not very important,” and 1.5% (15/1010) believed it was “Not at all important.”

Region also played a role in participants’ feelings about the disclosure of AI use, as respondents in the EU were less likely (50.0% of EU respondents, 209/418) than respondents in the US (71.4%, 357/500) or UK (69.6%, 64/92) to say that it was “Very important” for companies to disclose AI use, and more likely (41.6%, 174/418) than respondents in the US (22.8%, 114/500) or UK (21.7%, 20/92) to say that it was “Somewhat important” (*P*<.001).

## Discussion

### Principal Findings

Public exposure to generative AI has increased dramatically in recent years since the broad release of ChatGPT by OpenAI in November 2022. The results of our survey reflect the complex and occasionally contradictory views about generative AI held by the general public. Respondents demonstrated familiarity and confidence with the abilities of AI in general. However, despite this confidence, the overwhelming majority of respondents indicated that it was at least somewhat important for humans to be involved in the creation and review of clinical trial documents. Likewise, respondents reported having greater trust in clinical trial documents that had human involvement in their development. Perceived importance and trust in AI also varied by age and region, as older individuals and those from the US perceived human involvement as more important and were less trusting of AI-created documents lacking human involvement.

### Comparison With Prior Work

Our findings expand upon previous studies that have aimed to understand perceptions of AI use in healthcare. A recent survey of 1100 participants in Finland found that trust was driven more by attitudes towards technology in general than opinions about the use of AI in specific “use cases” [12]. In our study, respondents’ trust and confidence in AI in general were usually consistent with their attitudes about the particular “use case” of clinical trial documents. Interestingly, we also found that respondents’ reported trust of pharmaceutical companies and institutions conducting clinical research tended to be related to their trust of and confidence in AI. This suggests that trust of specific uses of AI may be linked not only to trust of AI in general, but to trust in institutions more broadly.

In general, participants who reported more familiarity with AI also reported greater confidence in its abilities. This relationship between familiarity and confidence in AI is consistent with a survey recently published by Reuters about trust in the use of AI in journalism [20]. This is also in line with prior research that has shown that people with previous exposure to AI-based tools were more likely to have favorable views towards AI use in health care [19].

Trust in AI is clearly complex and context-dependent, however. A 2021 systematic review of patient and public attitudes toward clinical AI found that AI is seen as both increasing and decreasing accuracy, depending on how well the AI has been trained in certain contexts [15]. This may shed light on apparently contradictory findings in our study, as 66.9% (676/1010) responded “Agree” or “Strongly Agree” that AI “always produces accurate and appropriate text and images,” while 67.0% (677/1010) responded that it is “Very important” for humans to be involved with developing clinical trial documents. This underscores that trust in AI is context specific. Respondents to our survey also overwhelmingly noted the importance of disclosing if and how AI had been used to produce clinical trial documents. This is in line with perceived importance of human involvement, and the risk of harmful “medical disinformation” due to AI that has been described in the literature [21].

Demographics have been cited as an important factor driving trust in AI in healthcare settings [12]. However, different studies have led to different conclusions about demographic effects [19], suggesting that the effect of demographics may be specific to context or specific patient populations. Our results suggest that experience with AI and confidence in the accuracy of AI are functions of age, ethnicity and geography. Respondents who were younger, non-White and/or Hispanic, or European were more likely to have used AI and be confident in it compared to older, White non-Hispanic, or US-based respondents. Interestingly, respondents in the EU were more likely than respondents in the US to say that human involvement and disclosure of AI use is “Somewhat important,” and less likely to say that it is “Very important” in the context of patient- and public-facing clinical trial documents. This difference is echoed by a recent Pew survey that found that among all countries surveyed, the US had the highest percentage of respondents (50%) who were “more concerned than excited” about AI [22]. Respondents to the Pew survey also tended to trust the EU more than the US to regulate AI effectively [22].

### Limitations

This study had some limitations that should be considered. While researchers evaluated face validity before distribution and determined the survey instrument had high face validity, the survey instrument was not validated. Survey responses were obtained from panel-based convenience samples. Respondents were individuals who self-identified as individuals seeking health-related information and elected to receive email communications and survey invitations. Thus, results may not reflect the perspectives of the general population due to sampling bias, especially among those who did not speak English (in particular among European countries) and those with limited access to the internet or who were unwilling to respond to online solicitation. It should also be noted that terms like “familiarity” and “trust” were self-reported and not defined more specifically within the survey. Additionally, the survey item “AI always produces accurate and appropriate text and images” asked about two ideas (accuracy and appropriateness) but only allowed respondents to provide one response.

### Conclusions

Our findings suggest that while patients reported familiarity with and confidence in the capabilities of AI, familiarity and confidence alone did not necessarily translate into trust. Respondents emphasized the continuing need for human oversight in the preparation of patient-facing clinical trial documents and placed importance on transparency regarding the use of AI. Trust was lowest when AI was used without human involvement and highest when humans worked independently, underscoring the enduring desire for human judgment in research communication. These findings suggest that the responsible integration of AI into the development of clinical trial lay summaries requires both meaningful human review and clear disclosure of AI involvement. Together, these elements form the foundation for maintaining patient and public trust in clinical research communications.

## Supplementary material

10.2196/76547 Multimedia Appendix 1 Survey questionnaire.

10.2196/76547 Multimedia Appendix 2 Experience using AI.

## Abbreviations

AI: artificial intelligence
CISCRP: Center for Information and Study on Clinical Research Participation
EU: European Union
ICMJE: International Committee of Medical Journal Editors
TAM: Technology Acceptance Model
UK: United Kingdom
US: United States
UTAUT: Unified Theory of Acceptance and Use of Technology
