# Supplementary material for: Patient Perceptions on the Use of Artificial Intelligence in Creating Clinical Research Documents: Survey Study
Source: JMIR AI. 2026 Jun 22;5:e76547. doi: 10.2196/76547 (PMC13286328; doi:10.2196/76547)
Supplement: Multimedia Appendix 1 [file ai-v5-e76547-s001.docx]

Thank you for taking the time to participate in this survey about Artificial Intelligence (AI)! This survey is conducted by the Center for Information and Study on Clinical Research Participation (CISCRP), an independent non-profit organization.

AI is at the forefront of technology today. AI is designed to understand and learn from input to create useful text, images, and more. One example you may have heard of is ChatGPT, created by a company called OpenAI, but there are many different companies creating different types of AI for different purposes. One possibility is for pharmaceutical companies to use AI to write patient- and public-facing clinical trial documents, such as brochures, informed consent documents, study results summaries, and more.

Your opinions are very important, as they will help us better understand what people think about using AI and guide us in how to use AI in the development of patient- and public-facing clinical trial documents. The survey will take about 10 minutes to complete.

Your identity and individual responses are confidential – researchers will not be able to access personal documents about you and will only be reporting the results combined across all people who respond.

1. What is your age? [Open text box]
2. Which country are you located in? [Select from drop down]
3. Do you have a medical condition? [Yes/No]
4. Which of the following classification groups does your condition/disease fall into?  *(please select best option from list; if multiple conditions – select all that apply)* [Select from: Allergies; Arthritis; Blood; Cancer; Diabetes; Endocrine/Hormone; Eye; Female Sexual Health; Gastrointestinal; Headache/Migraine; Heart/Cardiovascular; Immune Condition; Infection; Kidney/Bladder; Lung; Male Sexual Health; Mental Health; Metabolism; Musculoskeletal; Neurology; Pain; Skin; Sleep; Other – Write In.]
5. Have you ever participated in (i.e. joined or enrolled) a clinical trial, also known as a clinical research study? [Yes/No/Don’t remember/don’t know]
6. In general, how much trust do you have in pharmaceutical companies or academic institutions conducting clinical research studies? [A lot/Some/A little/None]
7. Which of the following, if any, clinical trial documents have you seen? *(select all that apply)* [Select from: Informed consent document – A document that includes information about a clinical trial to help a person decide if they want to participate, including the possible risks and benefits of participating; Clinical trial protocol synopsis - A document that includes information about how a trial will be performed; Clinical trial publication summary – A document that includes information about the results of a clinical trial published in a medical journal; Informational brochure about clinical trials; Clinical trial results summary – A document that includes information about the results of a clinical trial; Informational brochure about a medical condition; Other – Write In; None of the above]
8. How familiar are you with Artificial Intelligence (AI), in general? [Very familiar/Somewhat familiar/Not very familiar/Not at all familiar]
9. Artificial Intelligence (AI) is a technology that allows computers to perform more complex tasks that historically only a human could do, such as reasoning, making decisions, or solving problems. Have you ever used AI? [Yes/No]
10. If yes, please specify how you have used AI. [Open text box]
11. AI always produces accurate and appropriate text and images. [Strongly agree/Agree/Disagree/Strongly disagree]
12. How many pharmaceutical companies and academic institutions, if any, do you think are currently using AI to write patient- and public-facing clinical trial documents? [A lot/Some/A little/None]
13. Please rate the following questions based on how much you would trust each of the following types of documents:
    1. If you knew a clinical trial document was written using AI, without any humans reviewing the document, how much would you trust the document? [A lot/Some/A little/None]
    2. If you knew a clinical trial document was written only by humans, how much would you trust the document? [A lot/Some/A little/None]
    3. If you knew a clinical trial document was written using AI, with humans reviewing the document, how much would you trust the document? [A lot/Some/A little/None]
14. How important is it for humans to be involved in the review and development of public- and patient-facing clinical trial documents? [Very important/Somewhat important/Not very important/Not at all important]
15. How important is it for pharmaceutical companies and academic institutions to make it clear if and how they have used AI in public- and patient-facing documents? [Very important/Somewhat important/Not very important/Not at all important]
16. How interested would you be, if at all, to read clinical trial documents that had been created at least in part by AI? [Very interested/Somewhat interested/Not very interested/Not at all interested]
17. Do you think that AI would increase or decrease spelling and grammar errors when creating a public- and patient-facing clinical trial document? [Increase/Decrease]
18. Do you think that AI would increase or decrease data errors when creating a public- and patient-facing clinical trial document? [Increase/Decrease]
19. What, if any, fears do you have about the use of AI in creating public- and patient-facing clinical trial documents? [Open text box]
20. What value, if any, do you see in the use of AI in creating public- and patient-facing clinical trial documents? [Open text box]

Next, we would like to answer some questions about you.

1. What is your gender? [Male/Female/Transgender male/Transgender female/Gender variant/non-conforming/Prefer not to answer/Other – Write In.]
2. What is your ethnicity? [Hispanic or Latino/Not Hispanic or Latino/Prefer not to answer/Other- Write In.]
3. What is your race? (Select all that apply) [American Indian or Alaska Native/Asian/Black or African American/Native Hawaiian or Pacific Islander/White/Prefer not to answer/Other -Write In.]
4. What is the highest level of education you have completed? [Select from: No schooling completed, or less than 1 year; Nursery, kindergarten, and elementary (grades 1-8/primary education; High school/secondary education (grades 9-12, no degree); High school/secondary education graduate (or equivalent); Technical or trade school training; Some college/higher education (1-4 years, no degree); Associate degree (including occupation or academic degrees); Bachelor’s degree (BA, BS, AB, etc.); Master’s degree (MA, MS, MENG, MSW, etc.); Professional school degree (MD, DDS, JD, PharmD, etc.); Doctorate degree (PhD, EdD, etc.); Prefer not to answer]
