# Supplementary material for: Patient Perceptions on the Use of Artificial Intelligence in Creating Clinical Research Documents: Survey Study
Source: JMIR AI. 2026 Jun 22;5:e76547. doi: 10.2196/76547 (PMC13286328; doi:10.2196/76547)
Supplement: Multimedia Appendix 2 [file ai-v5-e76547-s002.docx]

|  | **Have you ever used AI? (n=1,010)** | | **P value** |
| --- | --- | --- | --- |
|  | “Yes”, % (n) | “No”, % (n) |  |
| **All respondents** | 63.6% (642/1010) | 36.4% (368/1010) | -- |
| **Gender** |  |  | <.001 |
| Male | 70.6% (357/506) | 29.4% (149/506) |  |
| Female | 55.6% (268/482) | 44.4% (214/482) |  |
| Other^a^ | 77.3% (17/22) | 22.7% (5/22) |  |
| **Race/Ethnicity** |  |  | <.001 |
| White, non-Hispanic | 58.3% (346/593) | 41.7% (247/593) |  |
| Non-White or Hispanic^b^ | 71% (296/417) | 29% (121/417) |  |
| **Country/region** |  |  | <.001 |
| US | 55.4% (277/500) | 44.6% (223/500) |  |
| UK | 64.1% (59/92) | 35.9% (33/92) |  |
| EU | 73.2% (306/418) | 26.8% (112/418) |  |
| **Clinical Trial Experience** |  |  | <.001 |
| Yes | 76.5% (186/243) | 23.5% (57/243) |  |
| No^c^ | 59.5% (456/767) | 40.5% (311/767) |  |
| **Education** |  |  | =.002 |
| No Bachelor’s degree^d^ | 59.8% (373/624) | 40.2% (251/624) |  |
| Bachelor’s degree or higher | 69.7% (269/386) | 30.3% (117/386) |  |
| *P* values are from chi square tests, Bonferroni correction | | | |

^a^Survey respondents who selected “prefer not to answer” were also included as “other” for subgroup analysis.

^b^Survey respondents who selected “prefer not to answer” or “other” were also included as “Non-White or Hispanic” for subgroup analysis.

^c^Survey respondents who selected “unknown” were also included as “no” for subgroup analysis.

^d^Survey respondents who selected “prefer not to answer” were also included as “no bachelor’s degree” for subgroup analysis.
